# Hydroperiod Influences Tadpole Growth and Development in the Endangered Littlejohn's Tree Frog ( *Litoria littlejohni* )

**DOI:** 10.1002/ece3.70829

**Published:** 2025-01-09

**Authors:** Nadine Nolan, Matt Hayward, Alex Callen, Kaya Klop‐Toker

**Affiliations:** ^1^ Conservation Science Research Group, School of Environmental and Life Sciences University of Newcastle Callaghan New South Wales Australia; ^2^ Centre for African Conservation Ecology Nelson Mandela University Gqeberha South Africa

**Keywords:** amphibian, conservation, habitat creation, hydroperiod, metamorphosis, phenotypic plasticity, tadpoles

## Abstract

Amphibians are among the most threatened vertebrate taxa globally. Their global decline necessitates effective conservation actions to bolster populations across both the larval and adult stages. Constructing man‐made ponds is one action proven to enhance reproduction in pond‐breeding amphibians. However, to achieve successful conservation outcomes, extensive knowledge about the ecology and behavior of the target species is required. In this study, we investigated how different hydroperiod regimes impacted the growth and development of *Litoria littlejohni* tadpoles. Over a 28‐week period, tadpoles were exposed to three hydroperiod treatments: constant high, declining, and constant low water levels. Weekly measurements of snout‐vent length, body mass, and Gosner stage were taken to assess treatment‐related changes. To determine whether different treatments affected locomotor performance, jump tests were conducted 3 weeks post‐metamorphosis. Individuals exhibited limited developmental plasticity in response to declining water, with a mean time to metamorphosis of 85.1 days ± 12.1. Comparatively, when *L. littlejohni* tadpoles were exposed to low water volumes, they were able to speed up development and reduce time to metamorphosis, with a mean time of 63.7 days ± 10.3. The speeding up of development had an apparent consequence for *L. littlejohni* . We found support for trade‐offs between rapid development and reduced morphometric measurements postmetamorphosis which resulted in reduced locomotive ability. Individuals from constant low water treatments exhibited an average total jumping distance of 171 cm ± 13.6 over 10 consecutive jumps, compared with 236 cm ± 17.3 in constant high and 210 cm ± 14.8 in declining treatments. Rapid larval development aids tadpoles in escaping suboptimal aquatic conditions, but its effects on locomotion may impact foraging efficiency and predator escape ability. Understanding developmental plasticity in threatened amphibians, especially in response to hydroperiod variations, is crucial for conservation programs, particularly under future climate change scenarios predicting increased drought and reduced hydroperiods in aquatic environments.

## Graphical abstract

Amphibians globally face significant threats, prompting the need for effective conservation strategies across various life stages. Investigating hydroperiod effects on *Litoria littlejohni* tadpoles, we exposed them to different water levels over 28 weeks. While developmental plasticity was absent under declining water, tadpoles exposed to low water volumes exhibited accelerated development with trade‐offs in post‐metamorphosis morphometrics and reduced locomotive ability.

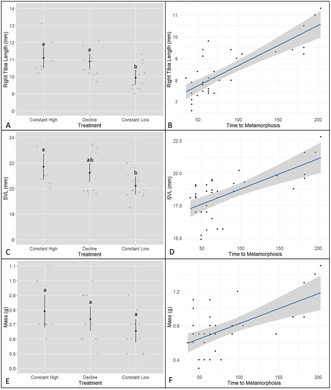

## 1 Introduction

Amphibian populations around the globe are in severe decline, with the International Union for the Conservation of Nature (IUCN) suggesting up to 51% are now threatened with extinction (IUCN 2023). Infection by the fungal pathogen *Batrachochytrium dendrobatidis* (Bd), which causes the disease chytridiomycosis in amphibians, is a key driver of these declines (Scheele, Pasmans, and Skerratt 2019; Speare et al. 1993; Wake 1998). The global threat to amphibians is further compounded by increases in anthropogenic habitat loss, degradation, and fragmentation, as well as climate change and the introduction of invasive species (D'Amore, Kirby, and McNicholas 2009; Harper, Rittenhouse, and Semlitsch 2008; Mansoor et al. 2022; Wilk, Donlon, and Peterman 2020). To mitigate these threats and help reverse the declines, conservation actions require all available tools including a combination of ex situ and in situ actions. Current literature suggests targeted methods that address multiple threats and bolster populations across both the larval and adult life stages for biphasic species may help improve conservation outcomes (Nolan et al. 2023). Furthermore, to improve conservation outcomes, Campbell et al. (2023) concluded that conservation actions that target species management should be prioritized, including research that has a greater focus on recovery, reintroduction, and *ex situ* conservation actions.

One conservation action that can greatly bolster reproduction in pond‐breeding amphibians is the creation of man‐made ponds (Mathwin et al. 2021). Man‐made ponds are usually small (1 m^2^ to about 5 ha) dams or shallow waterbodies with permanent or ephemeral hydroperiods (Céréghino et al. 2010; Rannap, Lõhmus, and Briggs 2010). For amphibian species that are biphasic and readily breed in such environments, pond creation may prove a promising conservation strategy that addresses two of the major threatening processes—anthropogenic habitat loss and droughts driven by climate change. Pond creation has proven effective for a range of amphibian species (Ashpole, Bishop, and Murphy 2018; Lambert et al. 2021; O'Brien et al. 2021; Vignoli et al. 2021), however, success rates are variable, and this may be linked to pond hydrology (Klop‐Toker et al. 2016; Valdez et al. 2019; Vasconcelos and Calhoun 2006). Therefore, to achieve successful conservation outcomes involving pond creation, extensive prior knowledge about the ecology, behaviour, and life history of the target species is required, particularly how they respond to variable hydroperiods (Mathwin et al. 2021).

There are both benefits and drawbacks associated with permanent and ephemeral ponds for biphasic amphibians that require aquatic habitat for breeding. Species‐specific ecological requirements may influence breeding choices between permanent and ephemeral ponds, with each habitat type having its own trade‐offs. Permanent ponds provide a stable aquatic environment that is beneficial to amphibian species with long larval development stages (Wilbur and Collins 1973). These ponds also provide suitable breeding habitat all year round, servicing multiple species with different breeding cycles (Babbitt 2005). However, as permanent waterbodies are the most likely to be colonized by fish, permanent ponds have the potential for high predation rates on eggs and tadpoles due to the presence of aquatic predators (Mathwin et al. 2021; Mogali, Saidapur, and Shanbhag 2017, 2016). Many studies show the negative effects of predatory fish on amphibian species (Klop‐Toker et al. 2018; Porej and Hetherington 2005; Walston and Mullin 2007; Watling, Hickman, and Orrock 2011), with these impacts so great that adults of some species avoid breeding in aquatic environments where predatory fish are present (Binckley and Resitarits 2003; Hopey and Petranka 1994). As species richness increases in permanent ponds, competition for resources, such as food and refuge also increases (Ramalho et al. 2021; Simpkins et al. 2014; Walls and Williams 2001). Ephemeral ponds, on the other hand, offer lower predation risk due to their shorter aquatic period (Mathwin et al. 2021; Zedler 2003). As ephemeral ponds generally completely dry out each year, many aquatic predators, such as fish and aquatic invertebrates, are eliminated (Holgerson et al. 2019; Jocqué, Graham, and Brendonck 2007; Pollard et al. 2017). In addition, ephemeral ponds can have reduced resource competition given there is a narrow window of opportunity for other species to establish themselves. However, ephemeral ponds are associated with shorter aquatic phases, reducing breeding potential (Baldwin, Calhoun, and deMaynadier 2006; McCaffery et al. 2014) and increasing desiccation risk for some amphibian species (Brady and Griffiths 2000; Richter‐Boix, Tejedo, and Rezende 2011). This is especially true for amphibians that have slow development times and do not demonstrate phenotypic plasticity in response to changing hydroperiods (Richter‐Boix, Tejedo, and Rezende 2011).

Some amphibian larvae demonstrate developmental plasticity and can alter the time it takes them to reach metamorphosis and leave the pond environment (Gomez‐Mestre et al. 2010; Mogali, Saidapur, and Shanbhag 2011). This change has been recorded in direct response to environmental conditions such as temperature, hydroperiod, and density of larvae (Amburgey et al. 2012; Plenderleith et al. 2019; Ren et al. 2021; Ruthsatz et al. 2018). However, the response to different environmental cues is highly variable among species possibly resulting from evolutionary differences and life histories. The physiological effects of different hydroperiod regimes on growth and development are relatively unknown for many threatened amphibian species. In the handful of studies that have examined the physiological effect of water permanence and ephemerality, results have varied. In the temperate frog *Rana pipiens* , Brannelly et al. (2019) found negative effects of a shortened hydroperiod on growth and development, with animals from fast and moderate drying regimes metamorphosing at a smaller size and having lower survival rates. Furthermore, Brannelly et al. (2019) reported sex‐specific disparities in larval period, with males undergoing metamorphosis faster than females in fast‐drying regimes. Le Sage et al. (2022) examined the effects of pond drying in three species of leopard frog ( *R. pipiens* , *R. sphenocephala* , and *R. chiricahuensis* ), and found that developmental plasticity in response to drying was rare. Le Sage et al. (2022), however, did note the exception in one southern population of *R. sphenocephala* that exhibited shorter larval periods and developed a more severe *Bd* infection, indicating a potential trade‐off between surviving pond drying and allocating resources to combat pathogens.

The endangered Littlejohn's tree frog ( *Litoria littlejohni* ) is endemic to New South Wales (NSW), Australia, and is only found in small, isolated populations that display high rates of inbreeding (Mahony et al. 2020; Stock et al. 2023). As a result of increased inbreeding within populations, combined with previous declines caused by *Bd*, habitat loss caused by the Black Summer bushfires in 2019/2020, and mining impacts (Klop‐Toker et al. 2021), concerns have been raised about the future persistence of this frog. A number of concurrent conservation efforts are underway to secure its persistence, including the creation of ponds as additional breeding sites within existing occupied habitat. This is considered worthwhile a*s L. littlejohni* breeds in isolated man‐made ponds and within‐stream pools with still or very slow‐flowing water that are absent of predatory fish and do not experience significant stream flow (Klop‐Toker et al. 2021).

A greater understanding of the physiological response of *L. littlejohni* to different hydroperiods is required to optimize conservation outcomes using the created habitat. Developmental plasticity in response to changing hydroperiods may be highly beneficial to *L. littlejohni* tadpoles, increasing the chance of survival during periods of hot and dry conditions (Cowan et al. 2014; Reddy, Perkins‐Kirkpatrick, and Sharples 2021). However, the consequences of speeding up development times in response to hydroperiod are poorly understood for many threatened species, making this study essential under today's climate crisis. To investigate how varying hydroperiod affects the developmental processes (such as growth rate and time to metamorphosis) of *L. littlejohni* tadpoles, and to assess their phenotypic plasticity in response to changing aquatic conditions, we conducted a controlled experiment. Tadpoles were housed under three distinct water level treatments: constant high water, constant low water, and declining water volume.

## 2 Methods

### 2.1 Animal Collection and Care

*Litoria littlejohni* tadpoles between Gosner stage 25 and 26 (Gosner, 1960) were collected from wild populations within Jilliby State Forest on the New South Wales Central Coast, Australia (33.08 °S, 151.36 °E, elevation 425 m). Tadpoles were acclimated to captivity over a 4‐week period before entering the experiment in December 2022. During the acclimation period, tadpoles were housed in clear plastic tanks (24 cm × 37 cm), each containing 10 L of 10% Holtfreter's Solution, a mixture of reverse osmosis water, 6.92 g NaCl, 0.2 g KCl, 0.14 g CaCl_2_·2H_2_O, and 0.82 g MgSO_4_·7H2O (Holtfreter 1931), with 10 tadpoles per tank. Throughout the experiment, tadpoles were housed in clear plastic tanks (24 cm × 37 cm) and maintained in a 10% Holtfreter's Solution, until Gosner stage 42 (determined by the appearance of the second forelimb). During the experiment, the laboratory temperature was maintained at 20°C (±1°). Weekly water temperature measurements were performed, and the water temperature was observed to be 21°C (±1°). Experimental UVB100 lighting maintained a 14:10‐h day‐to‐night ratio during the summer months and a 12:12‐h day‐to‐night ratio during the winter months.

Tanks were monitored daily to check tadpole health, food availability, and water level. A 50% water change was conducted on every tank once a week. Tadpoles were fed a diet of frozen baby spinach and freeze‐dried bloodworms *ad libitum*. The larval component of the experiment lasted 28 weeks. Once tadpoles reached Gosner stage 42, they were placed in clear, lidded tanks containing a water dish filled with 10% Simplified Amphibian Ringer. Metamorphs were kept under similar temperature (21°C ± 2°C) and UVB100 light conditions as the tadpoles and were fed five times a week on pin‐head crickets. All metamorphs were treated for chytridiomycosis with a 7‐day course of voriconazole.

### 2.2 Experimental Design

We conducted an experiment to test tadpole development and growth under three different water‐level treatments. We used a completely randomized design with three treatments and four replicates per treatment to assess the effects of varying water conditions on tadpole development. The treatments included declining water, where water levels were reduced by 0.5 L per week from an initial 10 L until reaching 3 L (approximately a 1 cm drop in water level per week), where it was maintained for the remainder of the experiment; constant low water, where a consistent water level of 3 L was maintained throughout; and constant high water, where a constant level of 10 L was maintained throughout the experiment. Constant high water was used as the control group in this study as it closely resembles the most natural conditions experienced by the *L. littlejohni* tadpoles in the wild. To compensate for water loss due to evaporation, tanks were monitored daily, and water levels were adjusted to maintain the specified treatment levels. Each plastic tank was marked with volume indicators in 0.5 L increments to facilitate accurate adjustments. Treatments were randomly assigned to experimental units, represented by tanks, with four tanks per treatment. Each tank housed five tadpoles, resulting in 20 tadpoles per treatment and 60 tadpoles across the entire experiment. Tadpoles were randomly selected from the rearing tank and assigned to experimental tanks, ensuring individuals of similar size to minimize size‐related bias. The tanks were randomly positioned within the laboratory to control for environmental variation, and to mitigate positional effects, the tanks' positions were rotated every seven weeks, totaling three rotations over the 28‐week experiment.

To measure tadpole development, we recorded the Gosner stage of individuals each week until the appearance of the second forelimb at Gosner stage 42. A hand‐held lens was used to determine the Gosner stage of each tadpole by looking at limb and mouthpart development. Tadpole growth was determined by measuring snout‐to‐vent length (SVL) and body mass once a week until the experiment concluded, or metamorphosis was reached (Gosner stage 42). Due to the rapid reabsorption of the tail once forelimbs emerge in tadpoles, all statistical analyses were conducted at Gosner stage 41 rather than stage 42 to ensure comparability of average mass between treatment groups. SVL was measured for each tadpole in hand with vernier callipers. The combined mass of all five tadpoles in each treatment was recorded with electronic digital scales (to the nearest 0.1 mg). The rate of metamorphosis was calculated as the proportion of tadpoles left in the experiment for each treatment.

To determine whether different treatments affected locomotor performance, a jump test was conducted 3 weeks after tadpoles fully metamorphosed (Gosner 46). To conduct the jump test, froglets were first dipped in water to wet their ventral surface and then placed on a clean, flat wooden surface. Wetting the frogs ensured that the landing position of each jump was marked with water. The frog was stimulated to jump by lightly touching their rear with a pen. A total of 10 jumps were performed by each froglet and the distance between each jump was measured with a ruler (to the nearest 0.5 mm). For each treatment group, we calculated the average total distance jumped and average longest single jump. To determine the average longest single jump, we identified the longest jump for each frog from the 10 recorded jumps and averaged these values within each treatment group. Each frog was weighed, and SVL and right tibia (RT) length were measured with vernier callipers immediately after each jump test.

### 2.3 Statistical Analysis

Generalized linear mixed models (GLMMs) were used to analyze various response variables across treatments, with likelihood ratio tests used to assess whether treatments had a significant effect. For total distance jumped, longest single distance jumped, post‐metamorphic RT length, SVL, and average mass, GLMMs were fitted using a Gaussian distribution with an identity link function, with treatment as a fixed effect and individual ID as a random effect. Tadpole average mass and tadpole SVL were modeled using a Gamma distribution with a log link function, treating treatment as the sole fixed effect. For models excluding Tank ID as a random effect, the Akaike Information Criterion (AIC) indicated better model performance. Time to metamorphosis, recorded in days, was modeled using a Poisson distribution, suitable for count data, with treatment as a fixed effect and individual ID and mass as random effects. A stepwise AIC‐based approach was applied to select the best‐fitting model.

Post hoc analyses were performed using pairwise comparisons between treatment groups for each model, with estimated marginal means and Tukey adjustments applied to control for multiple comparisons where necessary. Diagnostic tests conducted with the *DHARMa* package confirmed that model residuals met necessary assumptions. Power analyses using the *simr* package in R indicated sufficient power for time to metamorphosis (87%), metamorph RT length (96%), metamorph SVL (85%), and total jump distance (81%), all exceeding the 80% threshold. Tadpole SVL (78%) and largest jump distance (76%) had slightly lower power but still suggested a reasonable chance of detecting significant effects, while metamorph mass (55%) and tadpole mass (18%) had lower power, suggesting limited ability to detect smaller effects.

We conducted a Kaplan–Meier survival analysis to evaluate the rate of metamorphosis over time (in weeks) across the three treatment groups. Kaplan–Meier survival curves were generated, using metamorphosis events as the outcome variable. Log‐rank tests were performed to determine whether the survival curves differed significantly between treatments. These tests were conducted both overall and at specific weekly intervals to identify potential differences in the timing of metamorphosis. The log‐rank test calculates deviations between observed and expected metamorphosis events, accounting for the cumulative number of individuals at risk in each treatment group. Expected numbers of metamorphoses at specific time points were determined based on the overall probability of metamorphosis and the number of individuals at risk in each group. Differences between observed and expected events contributed to the chi‐square statistic, which was used to detect significant deviations between groups. Censored data included tadpoles that did not metamorphose by the end of the 28‐week study period, as well as the two tadpoles that died during the experiment.

An analysis of covariance (ANCOVA) was conducted to assess the relationship between RT length and total distance jumped across treatment groups while controlling for body mass and SVL. The ANCOVA model included RT length, body mass, SVL, and treatment as predictors, allowing for the examination of both the main effects and interactions between these variables. To assess the potential interaction between tibia size and treatment, an additional ANCOVA model was fitted that included the interaction term between RT length and treatment. The total distance jumped was treated as the dependent variable, and treatment as the categorical independent variable. RT length was used as the covariate to control for its effect on jumping performance. This analysis was performed to clarify whether the observed differences in jumping distance between treatments could be attributed to variations in tibia size.

To assess the relationships between time to metamorphosis and post‐metamorphic morphometric variables (RT length, SVL, and mass), simple linear regression models were used. For each model, the morphometric variable was treated as the dependent variable, and time to metamorphosis as the independent variable. The *R* ^2^ values represent the proportion of variance explained by the model, while the *p* values for the slope coefficients (*β*) were used to test the significance of the relationships. All statistical analyses were conducted using R version 4.4.1 (R Core Team 2023).

## 3 Results

### 3.1 Tadpole Development

**FIGURE 1:**
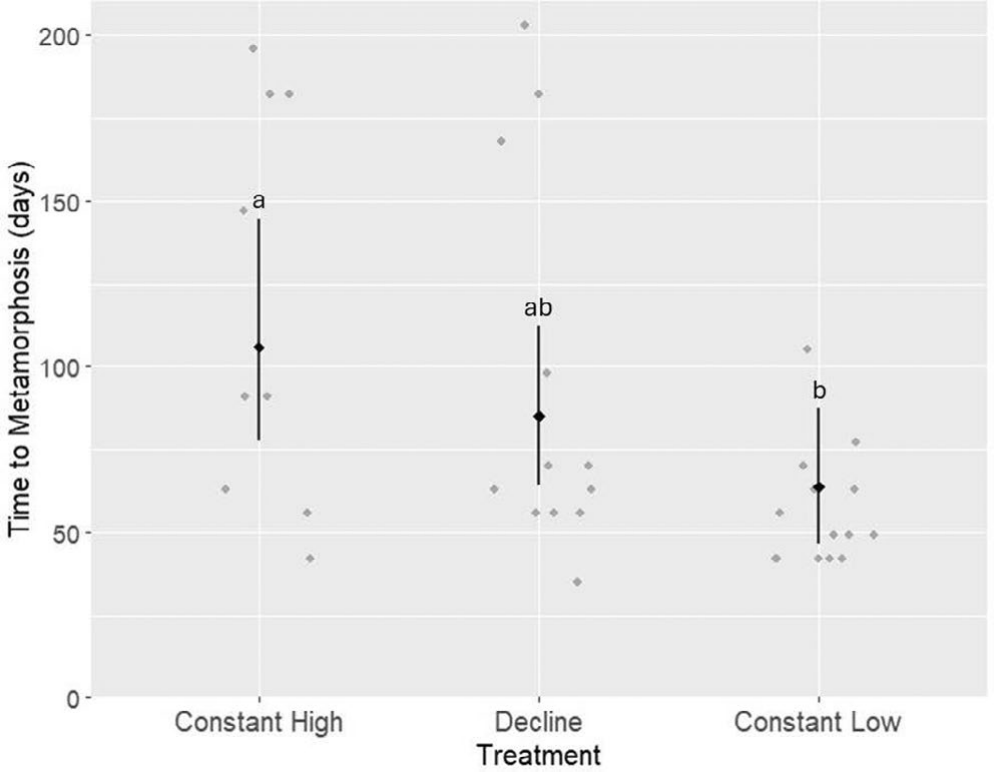
Time to metamorphosis for *Litoria littlejohni* across different treatment groups. The black diamonds represent the estimated means, while the black bars show the 95% confidence intervals (CIs). The grey points represent the raw individual data points for each treatment.

A total of 36 tadpoles metamorphosed within the designated experimental timeframe (28 weeks). At the end of the experiment, 22 individuals remained as tadpoles (37%). Two tadpoles (3%) died during the larval phase and therefore did not reach the end of the experiment. The likelihood ratio test indicated a significant effect of treatment on time to metamorphosis, *χ* ^2^(2) = 9.74, *p* < 0.05 (Figure 1). The constant high treatment group had a mean metamorphosis time of 105.9 days (±16.8 SE), the Constant Low group had a mean of 63.7 days (±10.3 SE), and the decline group had a mean of 85.1 days (±12.1 SE). Pairwise comparisons indicated that the constant high group took significantly longer to metamorphose than the constant low group (ratio = 1.66, 95% CI = 1.23–2.24, *p* < 0.05). There was no significant difference between the constant high and decline groups or between the constant low and decline groups. The random effects indicate that individual variability (ID, variance = 0.0814, SD = 0.2853) and mass (mass, variance = 0.1468, SE = 0.3831) contribute to the variation in total time to metamorphosis.

**FIGURE 2:**
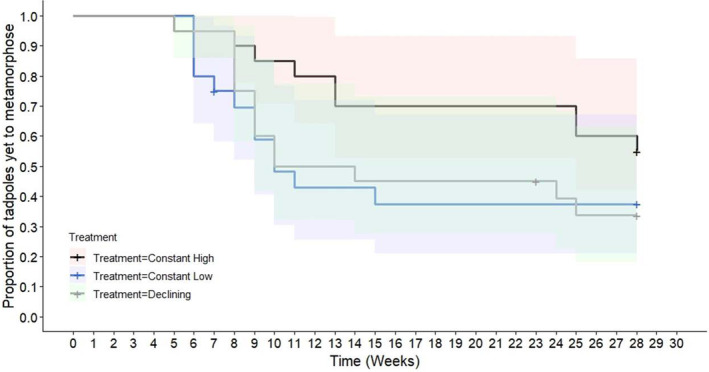
Kaplan–Meier survival curve showing the proportion of tadpoles yet to metamorphose over 28 weeks for three treatment groups: Constant high, constant low, and declining. Shaded bands represent 95% confidence intervals, and censoring is indicated by + symbols (at 7, 23, and 28 weeks). Two tadpole deaths were observed at weeks 7 and 23.

The proportion of tadpoles yet to metamorphose by week 28 ranged from 35% to 55%, with no significant differences between treatment groups over the entire 28‐week period (*χ* ^2^ = 3.1, df = 2, *p* = 0.2). However, at 7 weeks, the log‐rank test showed a significant difference between the treatment groups (*χ* ^2^ = 7.3, df = 2, *p* < 0.05). The declining treatment group deviated the most, with 1 observed metamorphosis compared with an expected 0.14, contributing heavily to the chi‐square statistic. The constant low group also showed a deviation, with five observed metamorphoses compared with an expected 5.88. After 7 weeks, no significant differences were found, with *p*‐values ranging from 0.3 to 0.9 in subsequent weeks, indicating similar metamorphosis rates among treatments after this initial divergence. This suggests early significant differences in metamorphosis rates for both constant low and declining treatments compared with constant high, though these differences did not persist in later weeks (Figure 2).

### 3.2 Tadpole Growth

**FIGURE 3:**
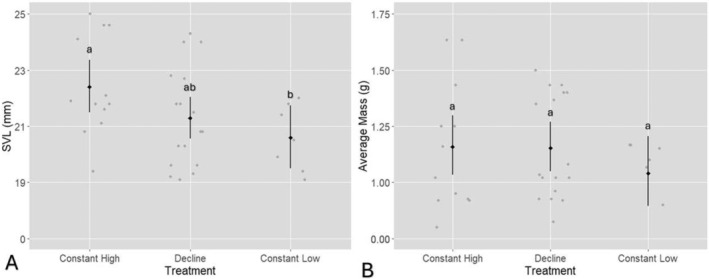
*Litoria littlejohni* tadpole morphometrics at Gosner stage 41: (A) Snout‐to‐vent length (mm) across treatment groups, and (B) average mass across treatment groups. The black diamonds represent the estimated means, while the black bars show the 95% confidence intervals (CIs). The grey points represent the raw individual data points for each treatment.

The likelihood ratio test indicated a significant effect of treatment on SVL at Gosner stage 41, *χ* ^2^(2) = 5.94, *p* = 0.05 (Figure 3A). The constant high group had an average SVL of 22.4 mm ± 0.48, while the decline group averaged 21.3 mm ± 0.38, and the constant low group had an average SVL of 20.6 mm ± 0.57. A pairwise comparison showed that the constant high group had a significantly greater SVL compared with the constant low group (estimated difference = 1.09 mm, 95% CI = 1.02–1.17 mm, *p* < 0.05). No significant difference was observed between the constant high and decline groups, and similarly, there was no significant difference between the decline and constant low groups. There was no significant effect of treatment on average body mass, as indicated by the likelihood ratio test, *χ* ^2^(2) = 1.50, *p* = 0.47 (Figure 3B). The constant low treatment resulted in an estimated reduction of mass by 10.8%, but this was not statistically significant (*p* = 0.259). Similarly, the decline treatment showed no significant difference in average mass compared with the constant high treatment (*p* = 0.96).

**FIGURE 4:**
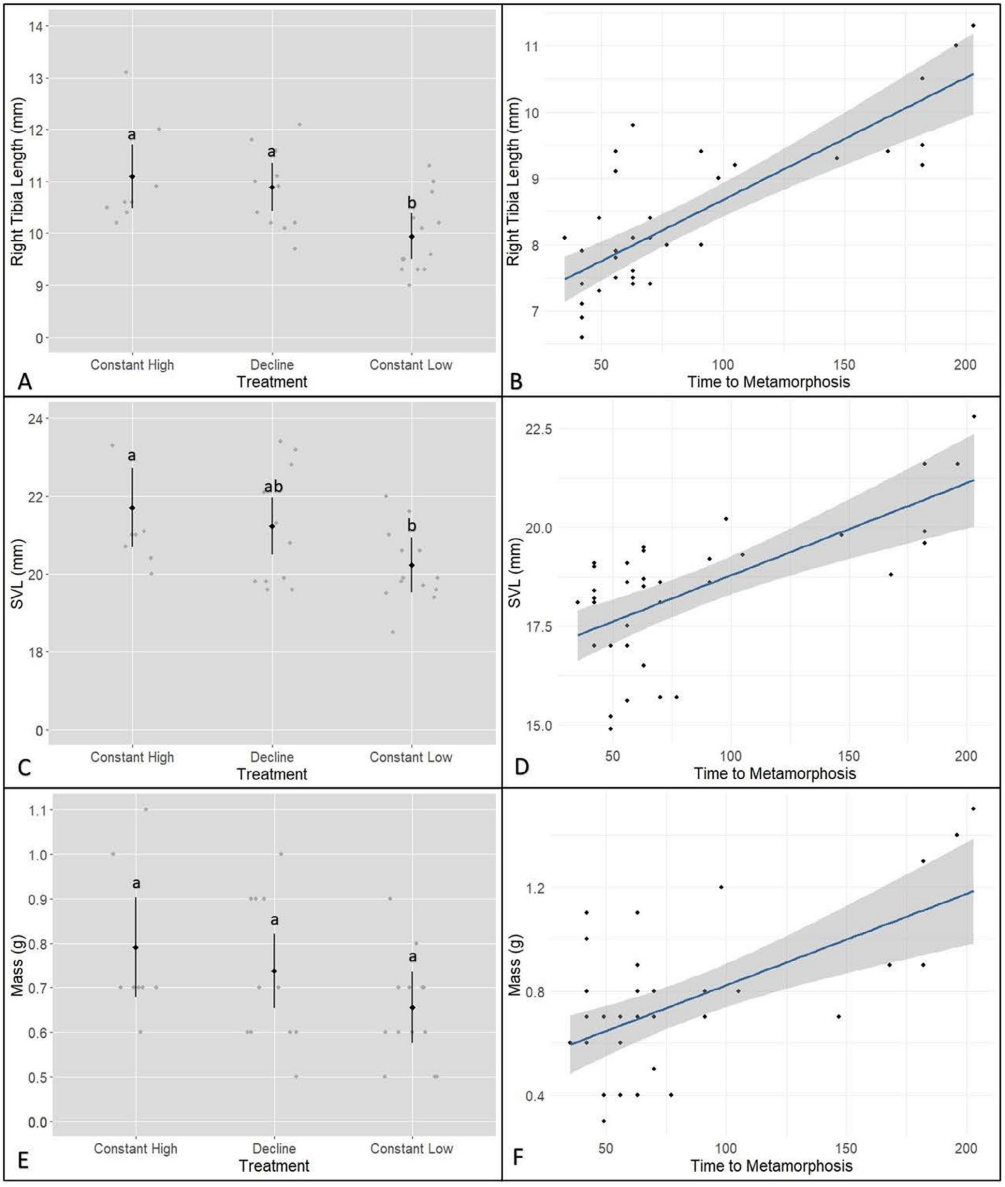
*Litoria littlejohni* frog morphometrics at the time of the jump test (Gosner stage 46): (A) right tibia length (mm) across treatment groups; (B) relationship between right tibia length and time to metamorphosis; (C) SVL (mm) across treatment groups; (D) relationship between SVL and time to metamorphosis; (E) mass (g) across treatment groups; and (F) relationship between mass and time to metamorphosis. The black diamonds represent the estimated means, while the black bars show the 95% confidence intervals (CIs). The grey points represent the raw individual data points for each treatment. The blue lines indicate linear fits with 95% confidence intervals.

The likelihood ratio test indicated a significant effect of treatment on average RT length at Gosner stage 46, *χ* ^2^(2) = 11.84, *p* < 0.05 (Figure 4A). The constant high treatment group had an average right tibial length of 11.10 mm ± 0.30, while the decline group averaged 10.89 mm ± 0.23, and the constant low group had an average tibial length of 9.95 mm ± 0.22. Pairwise comparisons revealed that the constant high group had a significantly greater tibial length compared with the constant low group (estimated difference = 1.15 mm, 95% CI = 0.24–2.07 mm, *p* < 0.05). Similarly, the decline group had a significantly longer tibial length compared with the constant low group (estimated difference = 0.95 mm, 95% CI = 0.24–1.65 mm, *p* < 0.05). No significant difference was observed between the constant high and decline groups. The likelihood ratio test indicated a significant effect of treatment on SVL at Gosner stage 46, *χ* ^2^(2) = 7.25, *p* < 0.05 (Figure 4C). The constant high treatment group had an average SVL of 21.7 mm ± 0.49, while the decline group averaged 21.2 mm ± 0.36, and the constant low group had an average SVL of 20.2 mm ± 0.34. Pairwise comparisons revealed that the constant high treatment group had a significantly larger SVL compared with the constant low group (estimated difference = 1.47 mm, 95% CI = −0.02 to 2.97 mm, *p* < 0.05). No significant difference was found between the constant high and decline groups, nor between the decline and constant low groups. There was no significant effect of treatment on average body mass, as indicated by the likelihood ratio test, *χ* ^2^(2) = 4.58, *p* = 0.10 (Figure 4E). The constant high treatment group had an average weight of 0.79 ± 0.05, the decline group averaged 0.74 ± 0.04, and the constant low group had an average weight of 0.66 ± 0.04. A pairwise comparison showed no significant differences between any of the treatment groups.

To investigate the potential causes behind the observed differences in post‐metamorphic morphometrics (RT length and SVL), we examined the correlation between time to metamorphosis and these traits. Time to metamorphosis was found to be significantly positively correlated with post‐metamorphic RT length (*R* ^2^ = 0.65, *β* = 35.40, *p* < 0.05; Figure 4B), SVL (*R* ^2^ = 0.45, *β* = 19.33, *p* < 0.05; Figure 4D), and mass (*R* ^2^ = 0.39, *β* = 109.99, *p* < 0.05; Figure 4F). These results suggest that longer developmental periods were associated with larger morphometric measurements post‐metamorphosis.

### 3.3 Post‐Metamorphic Locomotive Test

**FIGURE 5:**
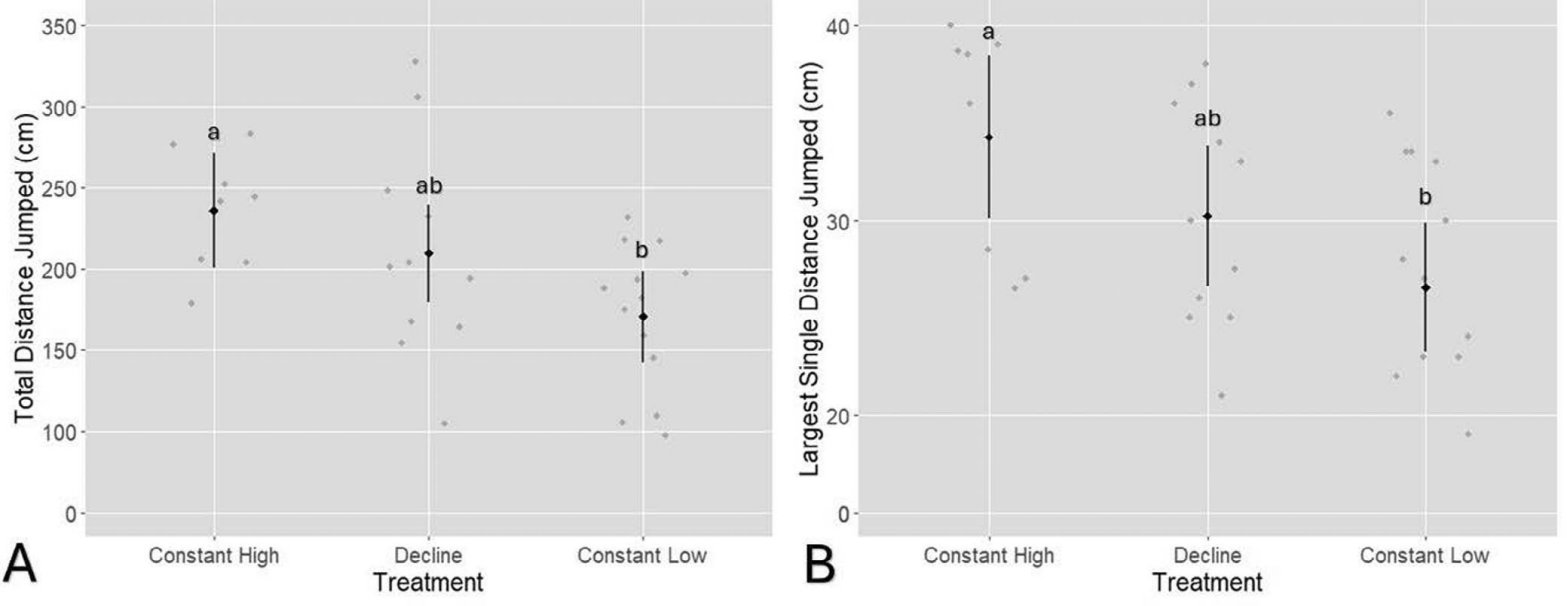
*Litoria littlejohni* post‐metamorphic Locomotive Test (Gosner stage 46): (A) total distance jumped (cm) across ten consecutive jumps and (B) the single largest distance jumped (cm) out of the ten jumps between treatments. The black diamonds represent the estimated means, while the black bars show the 95% confidence intervals (CIs). The grey points represent the raw individual data points for each treatment.

The likelihood ratio test indicated a significant effect of treatment on the average total distance jumped, *χ* ^2^(2) = 8.25, *p* < 0.05 (Figure 5A). The constant high treatment group had an average total distance jumped of 236 cm ± 17.3, while the decline group averaged 210 cm ± 14.8, and the constant low group had an average total distance jumped of 171 cm ± 13.6. A pairwise comparison revealed that the constant high treatment group had a significantly greater total distance jumped compared with the constant low group (estimated difference = 65.3 cm, 95% CI = 10.9–119.8 cm, *p* < 0.05). However, no significant difference was observed between the constant high and decline groups. Similarly, there was no significant difference between the decline and constant low groups.

The average longest single jump distance also differed between treatment groups, as indicated by the likelihood ratio test, *χ* ^2^(2) = 7.85, *p* < 0.05 (Figure 5B). The constant high treatment group had an average longest single jump distance of 34.3 cm ± 2.06, while the decline group averaged 30.2 cm ± 1.75, and the constant low group had an average longest single jump distance of 26.5 cm ± 1.61. A pairwise comparison revealed that the constant high group had a significantly greater longest single jump distance compared with the constant low group (estimated difference = 7.74 cm, 95% CI = 1.27–14.20 cm, *p* < 0.05). No significant difference was observed between the constant high and decline groups, and similarly, there was no significant difference between the decline and constant low groups.

There was a significantly positive relationship between RT length and total jump distance (*F*_(1,26)_ = 14.42, *p* < 0.05), suggesting that longer tibia length was strongly associated with greater jumping performance. However, neither body mass (*F*_(1,26)_ = 0.25, *p* = 0.62) nor SVL (*F*_(1,26)_ = 0.093, *p* = 0.76) had a significant effect on total jump distance. After accounting for these covariates, there was no significant difference between treatments in terms of total jump distance (*F*_(2,26)_ = 1.18, *p* = 0.32). When testing for interactions between treatment and RT length, the interaction effect was also non‐significant (*F*_(2,24)_ = 1.24, *p* = 0.31), indicating that the relationship between tibia length and jumping performance was consistent across treatments. Overall, these results suggest that while tibia size significantly influences jumping performance, treatment effects on total distance jumped were not significant once tibia length, weight, and SVL were controlled for.

## 4 Discussion

Water volume has a major effect on *L. littlejohni* larvae growth and average time to metamorphosis. Low water volumes were associated with smaller body size and a faster development time which is negatively correlated with body mass. Animals in the declining treatment group also experienced faster times to metamorphosis and a smaller body size at metamorphosis, but not to the same extent as animals in the constant low treatment, and these trends were not significantly different from either constant water level. This pattern suggests that *L. littlejohni* exhibits some developmental plasticity in response to gradual reductions in water levels, but it may not be as strong a signal on metamorphosis as low water levels. Because the temperature was kept constant and food was readily available, the trigger to metamorphose early for the animals in the constant low treatment was likely driven by density. Stress and toxins may accumulate during the early developmental stages (Gosner 25–26), potentially triggering early metamorphosis. This effect related to density may explain why tadpoles in the low water volume treatment group started metamorphosing early on in the experiment, suggesting that physiological plasticity is at play. An organism's physiological responses to stress can influence growth and development, making it a potential mechanism that contributes to phenotypic plasticity (Denver 2009; Maher, Werner, and Denver 2013). Environmental changes trigger thyroid hormone (TH) and glucocorticoids release in amphibians via the hypothalamic‐interrenal‐adrenal axis (Denver 1997a). THs regulate lipid metabolism, while corticosteroids work with THs to initiate metamorphosis (Denver 2009; Tata 2006).

### 4.1 The Influence of Declining Water Volume

In this experiment, *L. littlejohni* exhibited limited plasticity in their larval development in response to changing hydrological conditions. However, there is evidence of a partial response to declining water levels, with one individual metamorphosing as early as week five and the highest number of metamorphoses occurring in week eight, when water levels had decreased to 6 L. This shift in metamorphosis rate early in the experiment could be influenced by the reduced water volume and associated chemical stress, such as increased nitrogenous waste from metabolic processes. Although the outcomes observed in the declining treatment indicate partial developmental plasticity, where the species may detect and adjust to gradual changes in hydrological conditions but not as strongly as under extremely low water conditions. This response may reflect an adaptive strategy that balances developmental acceleration with maintaining growth potential, allowing tadpoles to mitigate risks associated with premature metamorphosis.

The fact that the declining treatment outcomes are not significantly different from either the constant high or constant low treatments suggests a nuanced response, where tadpoles prioritize flexibility under moderate environmental stress. On reaching a minimum body size required for metamorphosis, tadpoles can adjust the timing of this transformation by balancing growth opportunities against the risks of mortality in their larval habitat, a process regulated through the modulation of TH production, the primary driver of metamorphosis (Denver 2021). This intermediate pattern highlights that *L. littlejohni* is not entirely insensitive to declining water levels but lacks the pronounced developmental plasticity exhibited by other amphibian species capable of rapidly accelerating development in response to such conditions. This has important consequences for their continued survival under future climate change scenarios where drought is predicted to increase, reducing hydroperiods of aquatic breeding environments. Furthermore, longwall mining threatens one of the more southern populations found in NSW by reducing water availability within ponds (The Ecology Lab 2007). If *L. littlejohni* cannot adequately respond to declining water levels, the species may face an increased risk of incomplete larval development, leading to reduced recruitment and population declines.

Creating new ponds as a conservation action requires an understanding of the relationship between changing hydroperiods and developmental plasticity in tadpoles. Depending on how a species responds to different biotic and abiotic factors within a water body can help inform conservation managers when designing for pond size, shape, and depth. Our results show that *L. littlejohni* tadpoles have limited developmental plasticity when water volumes are declining, suggesting a partial ability to detect physical changes in water volumes over time and adjust their development accordingly. The degree of plasticity among pond‐breeding amphibians varies greatly (Rudolf and Rödel 2007; Ujszegi et al. 2022). Many species have the ability to accelerate their development in response to declining water (Marquez‐Garcia et al. 2009; Newman 1988; Perotti, Jara, and Ubeda 2011), however, most exhibit reduced growth and body mass at metamorphosis as a result of this faster development (Albecker, Strobel, and Womack 2023; Kehr et al. 2014). Our findings, however, conform with studies of several other pond‐breeding amphibians that do not rapidly increase their developmental rates when water levels decrease (Brady and Griffiths 2000; Morey and Reznick 2004; Richter‐Boix, Tejedo, and Rezende 2011).

Given the limited developmental plasticity observed in *L. littlejohni* in this experiment in declining water treatments, newly created ponds should vary in depth and size and priority should be given to deeper and more permanent ponds. In this experiment, tadpoles in declining water treatments were similar in SVL and mass to those in constant high treatments when they commenced metamorphosis (Gosner Stage 41). This trend remained consistent to the time individuals reached full metamorphosis (Gosner Stage 46). By creating deeper, more permanent ponds, *L. littlejohni* tadpoles would have an increased ability to reach metamorphosis, especially since this species has shown signs of overwintering (Klop‐Toker et al. 2021). This conclusion confirms recent study conducted by Wallace (2023) who found higher occupancy of *L. littlejohni* adults and tadpoles in ponds with depths between 0.5 and 1.5 m. Furthermore, this finding helps highlight the importance of swamp habitats to maintain water pond volume in the *L. littlejohni* populations in the southern Sydney bioregion.

### 4.2 The Influence of Low Water Volume

Contrary to the finding observed with declining water volumes, when *L. littlejohni* tadpoles were exposed to low water volumes, they were able to speed up development and reduce time to metamorphosis—an ability confirmed in many other amphibian species (Albecker, Strobel, and Womack 2023; Kehr et al. 2014; Loman 1999). This may indicate that *L. littlejohni* tadpoles are experiencing intraspecific crowding and chemical stress by detecting nitrogenous wastes from metabolic processes and adjusting their development rate accordingly (Schmuck, Geise, and Linsenmair 1994). Other studies have also found that tadpoles may speed up their development in response to increased ammonia and pH associated with low water levels (Gerlanc and Kaufman 2005; Morey and Reznick 2004). The build‐up of these chemicals may also trigger a stress response in tadpoles by activating the endocrine system through the release of TH and corticosteroids, accelerating metamorphosis (Denver 1997a, 1997b). In our experiment, despite a 50% water change with 10% Holtfreter solution in all treatments once a week, the build‐up of nitrogenous animal waste in the low water level treatment would have occurred at a higher rate compared with other treatment groups simply because of the higher population density, potentially leading to the observed effect. Because water volume and tadpole density could not be statistically separated in our experimental design, we are not able to identify which factor triggered reduced development times. To disentangle this effect, future studies could monitor oxygen, nitrate, and ammonium levels within changing hydroperiods and/or tadpole densities and examine tadpole development and growth.

### 4.3 Metamorphic Measurements and Locomotive Performance

The speeding up of development during the larval stage has an apparent consequence for *L. littlejohni* metamorphs. We found support for trade‐offs between rapid development and reduced morphometric measurements post‐metamorphosis. Reduced size linked to short larval periods have also been observed in several other amphibian species (Tejedo et al. 2010). In the northern leopard frog ( *Lithobates pipiens* ) under fast‐drying conditions, male individuals developed at a faster rate compared with females and metamorphosed at a smaller size (Brannelly et al. 2019). Individuals who developed faster have compromised immune systems, with lower antibody concentrations (Brannelly et al. 2019). In the South American toad ( *Rhinella arenarum* ), tadpoles with short larval periods metamorphosed at a smaller size (SVL) had fewer skin glands and had a thinner epidermis leading to physiological consequences post‐metamorphosis (Regueira et al. 2023). Conversely, in *Litoria ewingii* and *Litoria spenceri* tadpoles, separate studies found under crowded water conditions with high densities, tadpoles had slower growth rates, resulting in longer larval periods but smaller morphometrics post‐metamorphosis (Plenderleith et al. 2019; Sokol 1984). This indicates that speeding up development time alone is not the only driver in reduced morphometrics post‐metamorphosis. Other studies have attributed reduced morphometrics to conspecific chemical interference (Petranka 1989), resource competition (Álvarez and Nicieza 2002b; Steinwascher 1978), and temperature (Manasee, Weerathunga, and Rajapaksa 2020; Ren et al. 2021). Our findings, in contrast to other *Litoria* species, confirm the consequence of rapid larval development with reduced morphometrics post‐metamorphosis for *L. littlejohni* and while this is beneficial for tadpoles to escape suboptimal aquatic conditions it has flow on consequences for locomotive ability.

RT length correlated positively with total jump distance, and we found that individuals developing in low water volumes tended to have significantly shorter RT lengths and therefore shorter jump distances. In contrast, the intermediate outcomes observed in the declining treatment suggest less pronounced effects on post‐metamorphic measurements and locomotive performance compared with the constant low treatment. Locomotor ability is closely associated with a variety of ecological traits, including foraging efficiency, escape from predators, and energy budgets (Dickinson et al. 2000; Gomes et al. 2009). All of these features can impact growth, development, and survival later in life during the adult stage (Cooper Jr and Stankowich 2010; Lima and Moreira 1993). Other studies have found that exposure to different environmental variables (abiotic and biotic) during the early larval stage in amphibians can impact locomotor ability at metamorphosis (Álvarez and Nicieza 2002a; Callen et al. 2023; Clulow, Harris, and Mahony 2015). Post‐metamorphic green and gold bell frogs ( *Litoria aurea* ) reared as tadpoles in different salt concentrations exhibited significantly different jump distances (Callen et al. 2023) and Iberian painted frogs ( *Discoglossus galganoi* ) exposed to high temperatures and a plant‐based diet during the tadpole stage showed reduced jumping ability after metamorphosis (Álvarez and Nicieza 2002a). Given the reduced size and locomotor ability observed in our experiment for *L. littlejohni* and the potential consequence it can have on survival, further investigation into environmental impacts during the larval stage for threatened amphibians should be prioritized for conservation programs. These findings have important consequences for the survival of the species under changing environmental conditions, and for informing conservation actions such as the creation of man‐made ponds.

### 4.4 Conservation Relevance

Understanding developmental plasticity in threatened amphibians is important when planning conservation programs that involve early larval life stages. Biotic and abiotic environmental impacts experienced during larval development can have morphometric and, consequently, survival implications later in life. For the threatened *L. littlejohni* , variations in plasticity linked to hydroperiod are especially important to understand when habitat creation through man‐made ponds is used as a conservation tool to bolster populations, as is currently occurring. Tadpoles of *L. littlejohni* do not seem to detect physical decreases in water volume and, as a result, do not accelerate developmental rates. However, they may be sensitive to chemical changes in the water associated with reduced volumes and increased tadpole density, leading to accelerated development. However, accelerated development reduces size, which impacts locomotor ability and potentially, survival.

By understanding how changes in hydroperiod influence the developmental plasticity of Littlejohn's tree frog larvae, we can better predict how this species may respond to environmental changes that alter the hydroperiod of its breeding sites. These impacts include longwall coal mining and droughts caused by climate change that physically reduce water volumes in streams and ponds (Booth 2006; Klop‐Toker et al. 2021). Climate change is predicted to increase in frequency and intensity across *L. littlejohni's* distribution (Scheele et al. 2012; Sopniewski, Scheele, and Cardillo 2022) and will reduce the availability of surface water that relies on recharge by rainfall. Furthermore, longwall mining is occurring across the southern Sydney bioregion, an ecoregion that supports the three southern populations of *L. littlejohni* (Biosis 2016). Underground longwall mining can fracture the geology above the mine and cause a loss of both surface and sub‐surface water (Booth 2006; Hebblewhite 2020). In the Southern Sydney Bioregion, hanging swamps are a critical source of permanent water, which feeds water into many *L. littlejohni* breeding ponds. As longwall mining can drain hanging swamps, ponds that may have stayed watered during droughts may now face dewatering. Consequently, longwall mining and drought will cause freshwater habitats across the entire *L. litteljohni* distribution to become more ephemeral (Gorissen, Greenlees, and Shine 2017; The Ecology Lab 2007). The inability of *L. litteljohni* larvae to respond to declining water levels, and their reduced fitness when developing in small waterbodies, highlights how severe these two impacts may be on this species' long‐term survival.

Other studies have shown that *L. littlejohni* is highly sensitive to the presence of fish in breeding pools (Wallace 2023), making large, permanent ponds unsuitable breeding habitat. However, because our findings show that small, ephemeral ponds may impair long‐term survival by causing animals to be smaller at metamorphosis, we recommend habitat creation focus on ponds within a “goldilocks” zone of not too big and not too small. It appears that *L. littlejohni* need ponds that are not at risk of drying out during their prolonged development time, but that may dry out periodically to keep them fish‐free. Given that high tadpole density may contribute to shorter larval development times and smaller size at metamorphosis, we recommend constructing multiple ponds at a site to either accommodate egg‐laying by multiple females or allow females to distribute their clutches among different ponds, thereby reducing tadpole density per pond.

By creating clusters of man‐made ponds with variable hydroperiods, multiple amphibian species with various breeding cycles may also be bolstered (Beranek et al. 2021; Hartel, Bancila, and Cogalniceanu 2011). During times of increased extreme temperatures and drought events, like those predicted globally in the very near future, the number of ephemeral aquatic breeding habitats in the landscape will reduce. Consequently, amphibian reproduction will be increasingly dependent on the remaining permanent ponds and their connectivity across the landscape (Nolan et al. 2023). In addition, networks of ponds that offer variable hydroperiods protect against sub‐population extinction by enabling the rescue effect to influence recolonization via dispersal and immigration of juveniles or adults from an adjacent pond (Brown and Kodric‐Brown 1977; Griffiths 1997). Therefore, to bolster *L. littlejohni* and other amphibian populations in the wild, we recommend the creation of a network of man‐made ponds of diverse sizes and depths. An emphasis should be placed on ponds with longer hydroperiods, while also including a limited number of ephemeral ponds.

### 4.5 Limitations

One of the primary limitations of our study is the relatively small sample size, which was constrained by the fact that *L. littlejohni* is a threatened species. Due to its threat status, our scientific license only permitted the collection of a limited number of individuals for experimentation. This limitation affected the statistical power for variables like tadpole mass and metamorph mass. While we ran power analyses on all response variables in models, which indicated high power for most analyses, tadpole mass, and metamorph mass had lower power, potentially limiting our ability to detect smaller effects in these variables. This constraint should be considered when interpreting the results.

We originally aimed to include both high‐ and low‐temperature treatments in our experiment to comprehensively examine the effects of hydroperiod and temperature on larval development. However, when tadpoles were transferred to temperature‐controlled cabinets at the start of the experiment, we observed unexpected fatalities, leading us to abandon this aspect of the study. We suspect that noise and/or vibration within the cabinets may have contributed to these fatalities, as previous studies have shown that such environmental factors can adversely affect amphibians (Ferrie et al. 2014; Heatwole and Sullivan 1995; Tennessen, Parks, and Langkilde 2014; Vandenberg, Stevenson, and Levin 2012). Nevertheless, it is important to note that other studies have successfully used temperature‐controlled cabinets without such adverse effects (Callen et al. 2023; Enemar 1978; Ujszegi et al. 2022; van Uitregt et al. 2016), suggesting that our findings may have been influenced by specific conditions or sensitivities unique to our study species.

## Author Contributions

**Nadine Nolan:** conceptualization (supporting), data curation (lead), formal analysis (lead), investigation (lead), methodology (lead), project administration (lead), writing – original draft (lead), writing – review and editing (lead). **Matt Hayward:** conceptualization (supporting), funding acquisition (supporting), resources (equal), supervision (lead), writing – review and editing (supporting). **Alex Callen:** conceptualization (supporting), funding acquisition (supporting), methodology (supporting), project administration (supporting), resources (equal), supervision (equal), writing – review and editing (supporting). **Kaya Klop‐Toker:** conceptualization (lead), funding acquisition (lead), methodology (supporting), resources (equal), supervision (equal), writing – review and editing (supporting).

## Conflicts of Interest

The authors declare no conflicts of interest.

## Data Availability

The data underpinning the findings presented in this paper have been securely archived in The University of Newcastle's data repository, NOVA. Interested parties can access the dataset through the following link: https://doi.org/10.25817/6CXV‐PF94.
